# Hemodynamic Response to Interictal Epileptiform Discharges Addressed by Personalized EEG-fNIRS Recordings

**DOI:** 10.3389/fnins.2016.00102

**Published:** 2016-03-22

**Authors:** Giovanni Pellegrino, Alexis Machado, Nicolas von Ellenrieder, Satsuki Watanabe, Jeffery A. Hall, Jean-Marc Lina, Eliane Kobayashi, Christophe Grova

**Affiliations:** ^1^Multimodal Functional Imaging Laboratory, Biomedical Engineering Department, Montreal Neurological Institute, McGill UniversityMontreal, QC, Canada; ^2^Department of Neurology and Neurosurgery, Montreal Neurological Institute and HospitalMontreal, QC, Canada; ^3^Departement de Génie Electrique, Ecole de Technologie SupérieureMontreal, QC, Canada; ^4^Center of Advanced Research in Sleep Medicine, Hospital Du Sacre-CœurMontreal, QC, Canada; ^5^Centre de Recherches Mathematiques, University of MontréalMontreal, QC, Canada; ^6^Physics Department and Perform Center, Concordia UniversityMontreal, QC, Canada

**Keywords:** epilepsy, fNIRS, fMRI, hemodynamic response, interictal epileptiform discharges, EEG, MEG

## Abstract

**Objective:** We aimed at studying the hemodynamic response (HR) to Interictal Epileptic Discharges (IEDs) using patient-specific and prolonged simultaneous ElectroEncephaloGraphy (EEG) and functional Near InfraRed Spectroscopy (fNIRS) recordings.

**Methods:** The epileptic generator was localized using Magnetoencephalography source imaging. fNIRS montage was tailored for each patient, using an algorithm to optimize the sensitivity to the epileptic generator. Optodes were glued using collodion to achieve prolonged acquisition with high quality signal. fNIRS data analysis was handled with no a priori constraint on HR time course, averaging fNIRS signals to similar IEDs. Cluster-permutation analysis was performed on 3D reconstructed fNIRS data to identify significant spatio-temporal HR clusters. Standard (GLM with fixed HRF) and cluster-permutation EEG-fMRI analyses were performed for comparison purposes.

**Results:** fNIRS detected HR to IEDs for 8/9 patients. It mainly consisted oxy-hemoglobin increases (seven patients), followed by oxy-hemoglobin decreases (six patients). HR was lateralized in six patients and lasted from 8.5 to 30 s. Standard EEG-fMRI analysis detected an HR in 4/9 patients (4/9 without enough IEDs, 1/9 unreliable result). The cluster-permutation EEG-fMRI analysis restricted to the region investigated by fNIRS showed additional strong and non-canonical BOLD responses starting earlier than the IEDs and lasting up to 30 s.

**Conclusions:** (i) EEG-fNIRS is suitable to detect the HR to IEDs and can outperform EEG-fMRI because of prolonged recordings and greater chance to detect IEDs; (ii) cluster-permutation analysis unveils additional HR features underestimated when imposing a canonical HR function (iii) the HR is often bilateral and lasts up to 30 s.

## Introduction

The main goal of this investigation is to study the hemodynamic response (HR) to interictal epileptiform discharges (IEDs) using simultaneous recordings of Electroencephalography (EEG) and functional Near InfraRed Spectroscopy (fNIRS). IEDs are spontaneous and transient epileptic events occurring between seizures whose associated HR is usually exploited by combined EEG-fMRI to identify the epileptic generator (Laufs and Duncan, 2007; Gotman and Pittau, 2011; Heers et al., 2014; Pittau et al., 2014). During simultaneous EEG-fMRI investigation, scalp EEG data are used to monitor the occurrence of spontaneous IEDs, in order to characterize the BOLD response associated to these discharges. This technique is clinically useful to guide brain surgery and to achieve a better outcome in patients affected by drug resistant epilepsy (Zijlmans et al., 2007; Thornton et al., 2010, 2011; Pittau et al., 2012; An et al., 2013).

Simultaneous EEG-fMRI remains however very challenging. It is not available everywhere and requires specific data acquisition expertise. As any fMRI investigation, it also imposes strong immobility constrains and the acquisition time is usually short, typically 1 h (Cunningham et al., 2008; Gotman and Pittau, 2011; Huster et al., 2012; Chaudhary et al., 2013). Only patients with a relative high IEDs rate are scanned (An et al., 2013) and, in spite of this recruitment constrain, a percentage ranging between 23% (Pittau et al., 2012; An et al., 2013) and 48% (Aghakhani et al., 2006; Salek-Haddadi et al., 2006; Thornton et al., 2011) exhibit no EEG epileptic activity during the acquisition. For some of the remaining patients, no HR could be detected and the overall success rate is about 50% (Pittau et al., 2014). Moreover, standard EEG-fMRI analyses impose a canonical Hemodynamic Response Function (HRF) to occur after each IED. Although relaxing the constraints on the HRF provides higher sensitivity to the epileptic region (Kang et al., 2003; Bagshaw et al., 2004; Lu et al., 2006; Jacobs et al., 2008; Lemieux et al., 2008; Levan and Gotman, 2009; Storti et al., 2013), a full assessment of the HR time-course is very difficult with this technique and only a few attempts have been made (Benar et al., 2002; Salek-Haddadi et al., 2006; Masterton et al., 2010; Watanabe et al., 2014). Several studies using EEG-fMRI have also demonstrated that the HR is highly variable across patients and in some cases it is even possible to observe BOLD signal changes preceding rather than following IEDs (Hawco et al., 2007; Jacobs et al., 2009; Rathakrishnan et al., 2010; Pittau et al., 2011; Benuzzi et al., 2012), deactivations instead of the expected activations (Kobayashi et al., 2006b; Jacobs et al., 2007; Pittau et al., 2013) and HR clusters distant from the presumed IED generator (Heers et al., 2014).

Some fMRI limitations might be overcome by combining EEG with fNIRS, in particular the short recording time and the immobility constraints. This is a non-invasive method that measures the hemodynamic changes associated with brain activity (Villringer et al., 1994). Spatially distributed optic sources and detectors placed on the scalp emit and detect near infrared light. Because biological tissues are highly scattering and present low absorption of near infrared light, photons emitted by sources can go through the head following a diffusive process, reaching the cortex and being backscattered to the detectors. In the near infrared spectrum, Oxygenated and Deoxygenated Hemoglobins (HbO and HbR, respectively) are the two main optical absorbers in cerebral tissues. Hence the changes in optical density measured at two or more wavelengths allow estimating changes in HbO and HbR concentrations. fNIRS has some definite disadvantages compared to fMRI, such as spatial sensitivity restricted to cerebral cortex and lower spatial resolution, but allows long lasting acquisitions without strict immobility constraints, can be portable (Sawan et al., 2013; Jeppesen et al., 2015), is very suitable for studying sleep (Zhang and Khatami, 2015), owns high temporal resolution and is the only non-invasive technique able to disentangle fluctuations of both HbO and HbR. Altogether fNIRS recorded simultaneously with scalp EEG (EEG-fNIRS) should be feasible to study epileptic patients at bedside, regardless of their spiking rate or movements (Yucel et al., 2014).

In the last two decades many attempts have been performed to characterize the fNIRS HR to seizures (Villringer et al., 1994; Steinhoff et al., 1996; Adelson et al., 1999; Sokol et al., 2000; Watanabe et al., 2000, 2002; Haginoya et al., 2002; Buchheim et al., 2004; Munakata et al., 2004; Gallagher et al., 2008; Nguyen et al., 2012, 2013; Slone et al., 2012; Sato et al., 2013; Seyal, 2014; Yucel et al., 2014). However, seizures are infrequent and unpredictable and the question arises whether the same technique could also be applied to detect and characterize the HR to IEDs which are, conversely, relatively frequent and good marker of activity and location of the epileptic generator (Rosenow and Luders, 2001; Gotman, 2008).

This task is intrinsically challenging, because the hemodynamic response to IEDs is much weaker than the one to seizures (Kobayashi et al., 2006c) and fNIRS suffers from lower signal-to-noise ratio than fMRI (Cui et al., 2011). Recently Peng et al. have shown that EEG-fNIRS with large coverage and a standard analysis based on a fixed HRF achieves only low sensitivity and specificity in detecting the HR to IEDs (Peng et al., 2014).

We propose here a new strategy based on prolonged and personalized EEG-fNIRS acquisitions whose main aim is the detection and the time-course characterization of the HR to IEDs. The personalization is based on the identification of the epileptic focus and on the design of an optimal fNIRS montage specific for each subject. Since the epileptic generator identified on the basis of EEG or MagnetoEncephaloGraphy (MEG) source imaging of IEDs overlaps with the areas showing the most significant BOLD changes related to IEDs (Heers et al., 2014), we propose to use EEG/MEG source localization to optimally tailor the EEG/fNIRS investigation. In a previous study, we have indeed developed strategies to personalize the fNIRS montage to optimize its sensitivity to a predefined epileptic region, taking into account the positions of the EEG electrodes. We have showed that, compared to a standard montage, our personalization achieves a better sampling of the target region with a higher signal to noise ratio, using a lower number of optodes (Machado et al., 2014). The high sampling rate of fNIRS (20 Hz) and the relative high number of interictal discharges achieved because of prolonged acquisitions (up 4 h compared to 1 h of a standard EEG-fMRI) allow handling fNIRS data in an event-related design framework and avoiding to impose any a priori model of the HRF. For comparison purposes, a similar analysis was also performed on EEG-fMRI data from a subgroup of the same cohort.

## Materials and methods

### Patients

The study was performed in agreement with the Helsinki Declaration of 1975 (and as revised in 1983), was approved by the Research Ethics Board of the Montreal Neurological Institute and a written informed consent was obtained from all participants. Nine patients with focal epilepsy undergoing presurgical investigation at the Epilepsy Unit from the Montreal Neurological Institute and Hospital were studied (Table 1). Inclusion criteria were: (i) diagnosis of focal epilepsy; (ii) neocortical epileptic focus based on clinical history and seizure semiology, EEG features, and MRI data; (iii) presence of IEDs on telemetry; (iv) previous EEG-MEG recording for source localization to guide fNIRS individual montage. Exclusion criteria were: (i) patients whose epileptic focus location was unknown; (ii) concomitant cerebrovascular diseases.

**Table 1:** **Clinical information**.

**PA**	**Sex/Age**	**Epileptic focus**	**Anatomical MRI**	**EEG telemetry**	**EEG-MEG**		**EEG-fMRI results (GLM)**	
					**IEDs**	**Source loc**.	**IEDs**	**Activation/Deactivation**
PA 01	F/44	Left occipital	Non lesional	Posterior quadrant spikes, sharp-waves and brief burst of fast activity, synchronous, and asynchronous on both sides, more frequent on left side	Brief burst of rapid activity over the posterior quadrant regions of both sides, prevalent on the left side	Bilateral occipital sources, stronger on the left side, lateral occipital gyrus	Brief bursts of rapid activity over the left occipital cortex	Maximal activation left occipital lobe, lateral occipital gyrus. Albeit with lower *t*-values, other clusters were seen on both sides
PA 02	F/25	Right temporal	Non lesional	Spike and wave complexes and, mainly during sleep, burst of fast activity	Spike and slow waves	Right middle temporal gyrus	Spike and slow waves	Anterior aspect of right middle and superior temporal gyrus
PA 03	F/24	Right frontal	Non lesional	Bursts of fast activity with right frontal and temporal predominance during sleep; Bilateral and synchronous spike and Wave discharges with maximal amplitude over the frontal regions	Spike and slow wave	Right frontal	No IEDs	–
PA 04	M/53	Right occipital	Non lesional	Sharp and slow waves over the right posterior region, usually during sleep. Occasionally, he also presents right temporal sharp waves	Right posterior sharp waves	Right occipital, fusiform gyrus	No IEDs	–
PA 05	M/28	Left parieto-occipital	Non lesional	Very frequent and very low amplitude spikes over the PTO junction	Low amplitude spikes over the left parieto-occipital junction	Left Parieto—Occipital junction	Low amplitude spikes over the left parieto-occipital junction	–
PA 06	F/28	Right frontal	Non lesional	Bilateral and synchronous frontal spike and wave discharges, often prevalent in amplitude on the right side	Spike of the spike and wave complex	Right frontal focus	Spike and wave discharges	Activation with maximal *t*-value in the right frontal lobe, very widespread, including also the white matter
PA 07	F/21	Left frontal	Non lesional	Bilateral and synchronous SW discharges, possibly prevalent over the left frontal regions	Spike of the spike and wave complex	Left frontal	Spike and wave discharges	Multiple clusters of activations. Maximal *t*-value left parieto-temporal junction and left fronto-insular region
PA 08	F/29	Left temporal	Bilateral PNH	Left fronto-temporal spikes	Left fronto-temporal spikes	left temporal region	No IEDs	–
PA 09	M/35	Left temporal	Non lesional	Left fronto-temporal spikes, sometime organized in short bursts	Left temporal spikes	Left temporal pole	–	–

### Anatomical MRI and EEG/MEG data acquisition and analysis

#### Anatomical MRI

For each patient a 3D high resolution anatomical T1-MRI was acquired in a Siemens Tim Trio 3T scanner (T1W MPRAGE 1 mm isotropic 3D images, 192 sagittal slices, 256 × 256 matrix, TE 52.98 ms, TR 52.3 s) and was used for EEG/MEG source imaging, fNIRS optimal montage estimation and fNIRS 3D reconstruction.

#### EEG/MEG acquisition and analysis

Simultaneous EEG/MEG data (CTF-MEG system MISL, Vancouver, Canada; 275 MEG channels; 54 EEG electrodes) were recorded for about 1 h. IEDs were visually marked by two expert neurologists (G.P. and E.K). The location and spatial extent of the epileptic focus along patient's cortical surface was assessed applying the method reported in Heers et al. (2014, 2016) and using as inverse algorithm the coherent Maximal Entropy on the Mean (cMEM) method on average IED (Grova et al., 2006a; Chowdhury et al., 2013; Heers et al., 2014). For additional details the reader is referred to the Appendix A and to the tutorial of the Brain Entropy in space and time (BEst) toolbox included as a plugin of Brainstorm software (Tadel et al., 2011)^1^.

### Personalized fNIRS montage

The goal of the algorithm used to personalize the fNIRS montage was to identify the best source/detector arrangement on the patient's scalp that maximizes a priori the sensitivity of fNIRS measurements to a target Volume Of Interest (VOI) assumed to be the epileptic focus.

#### Definition of the target VOI

The EEG/MEG sources estimated at the IEDs peak along the cortical surface were thresholded at 70% of their maximum (Heers et al., 2014) and extrapolated into 3D volumes. To identify the correspondence between gray matter voxels and vertices of the cortical surface we used the anatomically driven non-overlapping interpolation kernels proposed in Grova et al. (2006b). This VOI and its homologous contralateral were considered as spatial priors for the optimal montage, as several studies reported bilateral fMRI hemodynamic responses to well lateralized interictal discharges (Kobayashi et al., 2006a; Gotman, 2008; Yu et al., 2009).

#### Estimation of the optimal fNIRS montage

**Figure 1:**
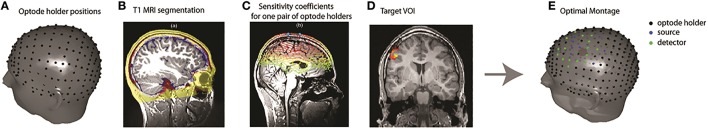
**Overview of the fNIRS optimal montage methodology. (A)** Optode holder positions based on the EEG 10/05 international system. **(B)** An anatomical head model was built from the classification of a T1 MRI into five tissue types. **(C)** Patient specific light sensitivity maps for each optode holder were estimated by Monte Carlo simulations. The sensitivity profiles of all possible pairs of optode positions were calculated. **(D)** Definition of the patient-specific target VOI, defined for instance from EEG-MEG source imaging results. **(E)** Optode montage optimization over the target volume using a branch and bound algorithm 4 sources (blue dots) 8 detectors (green dots) on each side.

The optimization method to tailor a patient-specific fNIRS optodes montage has been described by Machado et al. (2014) (Figure 1). A set of possible optode positions was computed on the patient skin mesh according to the EEG international standard system 10/05 system (Perdue et al., 2012). The 63 positions of the 10/10 system were reserved for EEG electrode placement, resulting in 248 possible optode positions distributed over the whole head surface (Figure 1). Patient-specific light sensitivity profiles for each possible pair of source/detector positions were estimated using a realistic subject specific anatomical head model built from an automatic segmentation of the T1 MRI into five layers (Collins et al., 1995). The goal of the optimization method was to find the best positions of sources and detectors among the set of potentials positions that maximize the global sensitivity (the sum of all sensitivity profiles for all source-detector pairs) in the target VOI. Due to the very large number of possible combinations, an exhaustive search was obviously non-feasible. This is the main reason why we formulated this optimization problem as a mixed linear integer programming problem (Land and Doig, 2010) and solved it using a branch and bound algorithm. The search space for the algorithm consisted in all the discrete indices of the standard 10/05 coordinates system as possible positions for sources and detectors. Additional functional constrains were implemented in the algorithm in order to ensure that the minimum number of sources and detectors required for the acquisition would be positioned and that two different optodes could not be placed at the same position (for further details on the description of this method and its validation, please refer to Machado et al., 2014).

### EEG-fNIRS acquisition

Simultaneous EEG and fNIRS data were acquired synchronizing an EEG system (Stellate Harmonie, Natus Medical Incorporated, USA) with a Brainsight fNIRS system (Rogue Research Inc., Montreal, Canada). EEG was acquired using 25 scalp electrodes placed according to the 10–20 (reference FCz) and 10–10 (F9, T9, P9, F10, T10, P10) systems (Ag/AgCl, sampling frequency 1000 Hz, offline bandpass 0.3–70 Hz). fNIRS acquisition was performed using eight sources emitting at both 690 and 830 nm and 16 photodetectors (sampling rate 20 Hz, maximal power 5 mW/wavelength). The exact position of electrodes and optodes on the skin of every patient was identified using the Brainsight 3D neuronavigation system (Rogue-Research Inc., Montreal, Canada). The procedure was the following: the T1 MRI of the subject and the predefined 3D coordinates of EEG electrodes and fNIRS optodes were entered into the system. The MRI and the patient's head were co-registered and all the EEG and optode positions were marked on the skin using a washable skin pen. Both EEG channels and fNIRS optodes were then glued to the skin using collodion. This adhesive mean, very common in the clinical practice for prolonged clinical EEG recordings, reduces motion artifacts and significantly improves data quality in prolonged fNIRS recordings (Yucel et al., 2014). The acquisition set-up was complemented by vertical and horizontal electro-oculogram, electrocardiogram, thoracic belt to monitor respiration, pulse-oximetry and video monitoring to identify clinical events. Data was acquired in a quiet room, with the patient sitting on a comfortable armchair, and being allowed to relax and to sleep. The recording lasted about 4 h divided in short 20-min runs.

### EEG-fNIRS analysis

All runs were analyzed. fNIRS signal quality was carefully checked using a homemade signal visualization tool. Noisy source-detector pairs were manually discarded on the base of absence of physiological activity in both 830 and 690 nm signals. Movement artifacts periods were marked by an experienced fNIRS user (AM) and corrected using spline interpolation (Scholkmann et al., 2010). Data was first bandpass filtered (0.005–0.3 Hz) in order to remove low frequency drifts signal components and cardiac fluctuations interferences, before being converted into optical density changes, considering as baseline reference the mean signal intensity obtained over the full run (Scholkmann et al., 2014).

EEG signals were re-referenced offline to bipolar and/or average reference montage. For each patient, we marked all the IEDs and checked that they had the same morphology and distribution as the ones recorded during telemetry and EEG-MEG. Events occurring close to identified fNIRS segments exhibiting movement artifacts (>5 s) were discarded from our analysis. To test the specificity of the hemodynamic response to IEDs, we also selected “control” events randomly chosen in IEDs-free periods. Selected EEG events were used to compute the average optical density at 830 and 690 nm time course relative to IEDs and control markers, for each fNIRS source-detector pair.

The subsequent analysis was performed using 3D diffuse optical tomography (DOT). The spatial support for 3D reconstruction consisted in gray matter voxels defined from the 1 mm resolution anatomical head model of each subject and constrained to the field of view investigated by optimal montage. Only the top 90% voxels to which the fNIRS montage was sensitive were considered, defining the so-called fNIRS field of view (FOV). DOT reconstructs Δ[HbO] and Δ[HbR] activity within the brain volume by solving an ill-posed inverse problem (Durduran et al., 2010). We considered a minimum norm inverse operator, for which the level of regularization was tuned within the Restricted Maximum Likelihood (ReML) framework (Abdelnour et al., 2010). The transformation from optical density to 3D variations in HbO and HbR concentrations was embedded within the inverse procedure using spectral decomposition of absorption coefficients. The level of regularization was tuned when localizing the averaged fNIRS signals from all IEDs and the same inverse operator was then applied to provide 3D fNIRS reconstruction for every single IED and control marker. The final output was an estimate around each single considered event (IED or control marker) of the time course of HbO and HbR concentration variations.

### Statistical analysis

We aimed at investigating the shape of the HR to IEDs, with no further modeling constraint. Consequently, usual generalized linear model approaches could not be considered. As prior information, we only assumed that the HR was expected to be temporally and spatially smooth. The analysis was performed in two steps. The first one was the identification of spatio-temporal clusters of HR significantly different from zero. The second step was the assessment of the specificity of the HR to IEDs, including the comparison to control events in the framework of a cluster/permutation approach.

#### Identification of activated voxels

We determined all voxels and time samples in which the mean HR over all IEDs was significantly different from zero (single sample *t*-test, alpha = 5%) and grouped them in clusters as soon as they were immediately adjacent in space (assuming 6-connectivity in 3D space) and occurring in consecutive time samples. Note that since the statistical detection approach consisted in identifying significant spatio-temporal clusters exhibiting activity sustained in time over a spatially extended region, there was not a significance threshold for the spatial extent of the clusters alone. To speed up computations, the clustering was carried out at a resolution of 2 mm per voxel and two samples per second and considering HbO signals only. Once a significant cluster was found for a specific region in space and time window, data from the original temporal and spatial resolutions was retrieved and projected on the cortical surface for visualization purposes. The shape of the hemodynamic response was defined as the average signal across all voxels of the cluster and all IEDs; its variability was reported as standard error across all IEDs.

#### Specificity of the detected fNIRS response

We performed a non-parametric permutation test with a Monte Carlo approach using the control events (Maris and Oostenveld, 2007). The null-hypothesis of the non-parametric test was that there is no difference between the strength of the clusters obtained from IEDs and control events, after having shuffled them through different permutations. The strength of each cluster was defined as the sum of the absolute *t*-values of all the voxels and time samples in the cluster. This definition combines the size of the cluster and its statistical significance (Maris and Oostenveld, 2007). The permutation test consisted on comparing the strength of the clusters obtained from the actual IEDs responses to the distribution of clusters strengths associated to the null hypothesis. This null distribution was obtained by pooling together the HR associated to every IEDs and control markers, and randomly selecting a subset of these signals. After having selected these permuted data, average signals and corresponding spatio-temporal clusters were estimated, using the same procedure described previously. Finally, the strength of each of these clusters, assumed to be drawn from the null distribution, was estimated. This procedure was repeated over 4000 permutations, and the significance of the spatio-temporal clusters associated to the actual IEDs was determined using a significance level of alpha = 5%. Note that this procedure accounts for multiple comparisons by design, and, since it is quite conservative, many clusters may not reach significance because of lack of statistical power.

### Comparison with EEG-fMRI data following a similar statistical analysis

For comparison purposes a similar statistical analysis was also performed on fMRI data of patients who also underwent simultaneous EEG-fMRI investigation. The recruitment for EEG-fMRI was independent from the inclusion/exclusion criteria of this study and was part of the multimodal evaluation in our Epilepsy center. This was often guided by clinical motivation, spike rate at telemetry (An et al., 2013) and specific research topics. EEG-fMRI data acquisition and standard GLM analysis is described in further details in the Appendix B and in Fahoum et al. (2012).

EEG-fMRI data was also analyzed using the same clustering and permutation analysis as for EEG-fNIRS. In order to compare EEG-fNIRS and EEG-fMRI, this analysis was applied only on the voxels belonging to the fNIRS FOV. To make sure we measured comparable signals in fNIRS and fMRI, the global signal was not regressed out of the fMRI measurements for this cluster analysis, because in fNIRS this global signal cannot be measured because of the limited FOV.

## Results

### General information

Nine neocortical focal epilepsy patients were recruited (Table 1). The anatomical MRI showed unremarkable findings for all the patients except PA08, who was affected by periventricular nodular heterotopia. The EEG-fNIRS acquisition lasted about 4 h for all the patients except for PA08, who was scanned for about 3 h. None of the patients reported any side effect or discomfort.

### EEG-fNIRS hemodynamic response

#### fNIRS hemodynamic response shape

**Figure 2:**
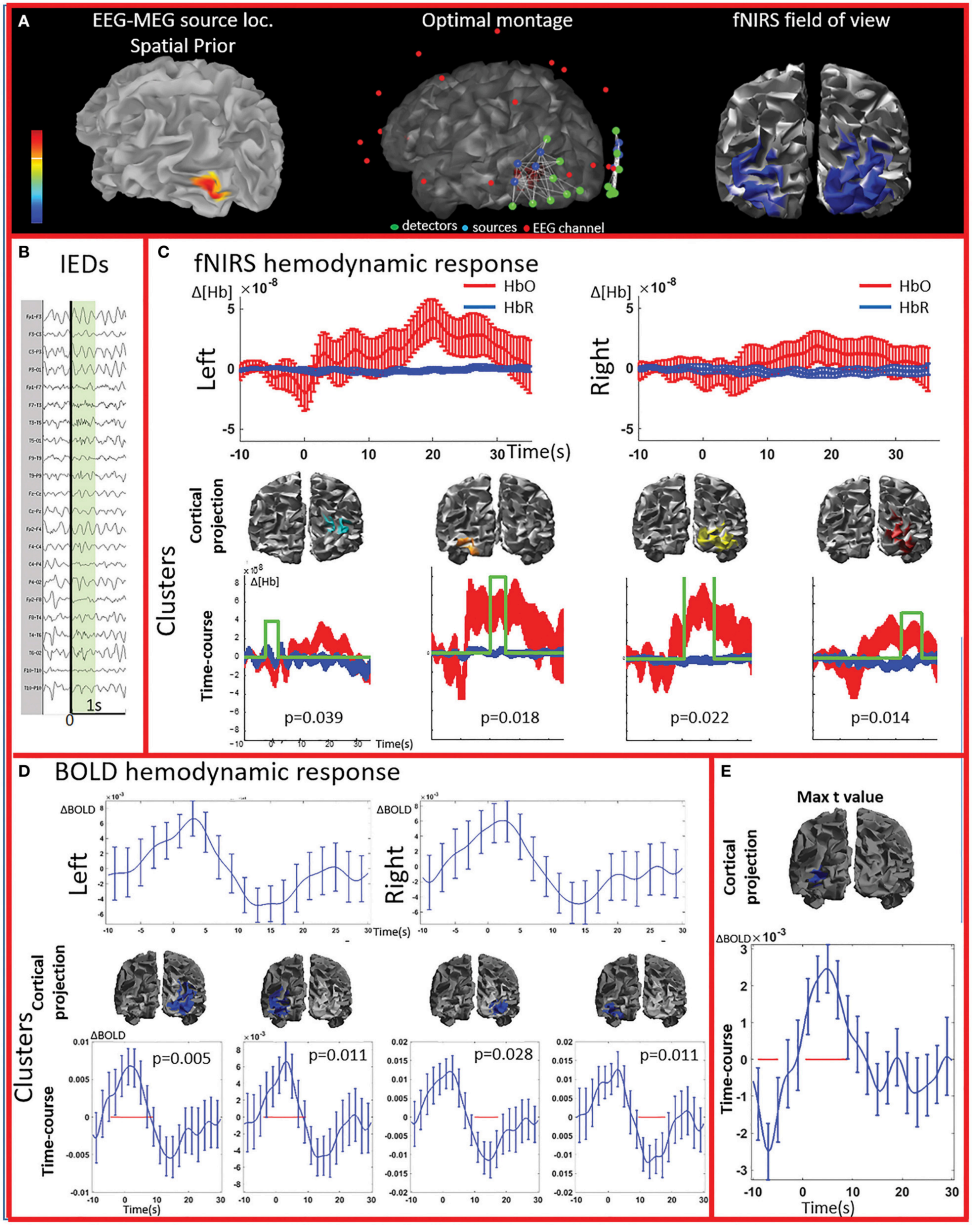
**Multimodal investigation of patient PA01 with left occipital epilepsy. (A)** Left occipital EEG-MEG source was used as a spatial prior to compute the optimal montage aimed at maximizing fNIRS sensitivity to the underlying epileptic region. **(B)** Short burst of fast activity in the beta range over the left posterior quadrant regions. **(C)** Upper line: fNIRS signal averaged (± standard error across epileptic events) over all voxels in the left and right fields of view. The left side was the most affected, but epileptic activity was also found over the right posterior hemisphere on multiple occasions. Lower line: clusters obtained from the comparison with control markers. For each item, the time-course refers to the average HbO and HbR changes in the cluster and the green line indicates when the hemodynamic response to IEDs was significantly different from the hemodynamic response to control events (x and y scales are the same for all the graphs). The estimated *p*-value of strength of each cluster is indicated on each graph. Significant clusters were present both on the left and right side but the one with highest amplitude was located in the left side. **(D)** Upper line: BOLD hemodynamic responses to IEDs for the left and right fields of view (averaged over all voxels ± standard error across epileptic events). To be noted, the response starts earlier than zero and lasts more than 20 s. Lower line: clusters showing a response to IEDs significantly different from the ones following the control markers. The blue line corresponds to the average bold signal in the cluster (± standard error across events). The red line indicates significativity. Clusters were found on both sides and showed a BOLD increase followed by a BOLD decrease. Note that the scale on the y axes are different across pictures and undershoots were showing the highest amplitude. Additionally, shape and duration of the hemodynamic response were not canonical. **(E)** The amplitude of the averaged hemodynamic response in the cluster exhibiting the maximal *t*-value from standard fMRI GLM analysis was actually lower than in brain areas identified by the cluster analysis in the fNIRS field of view.

**Figure 3:**
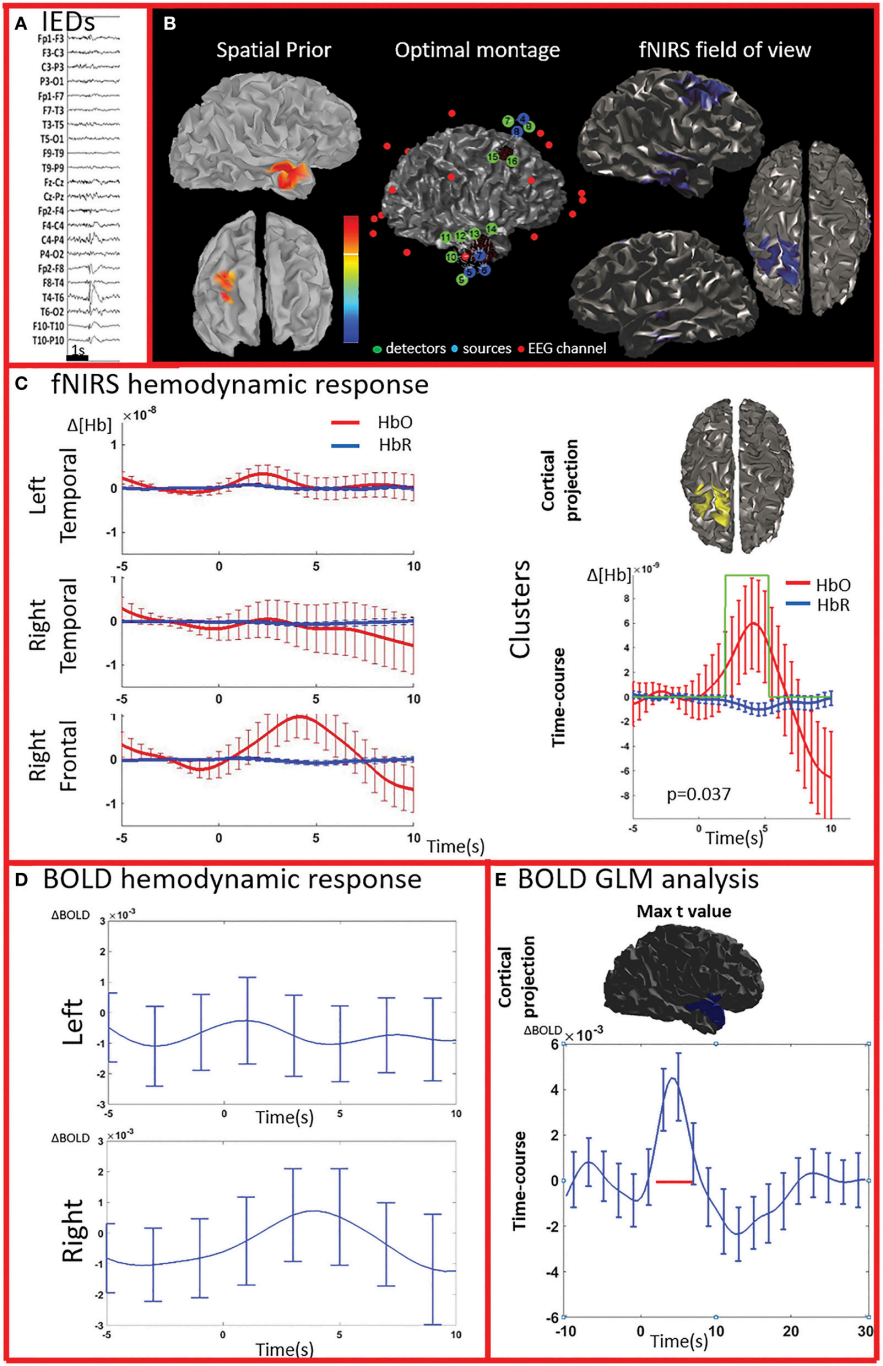
**Multimodal investigation of patient PA02**. Details regarding the organization of this figure are similar than those presented in Figure 2. **(A)** Spike and wave discharges over the right temporal region. **(B)** Left: Two EEG/MEG sources spatial were considered as spatial priors (i) a right temporal source from a recent EEG-MEG scan and (ii) a right frontal source, from a scan performed few years before. At the time of the investigation, however, the patient only showed IEDs over the right temporal regions. Because of low signal quality, the two fNIRS channels covering the left unaffected frontal prior were discarded from our analysis. **(C)** fNIRS average hemodynamic response detected on both sides, but of larger amplitude over the right/affected hemisphere. The only cluster exhibiting of hemodynamic activity significantly different from the one associated to control events was found in the right frontal region (right section of panel **C**). **(D)** The BOLD hemodynamic response in the fNIRS field of view was overall weak or absent. No cluster of significant difference vs. control events were found either in the temporal or frontal fNIRS regions. **(E)** After standard GLM analysis the significant cluster with maximal *t*-value was found in the right temporal pole. In this region the time-course of the BOLD signal consists of a strong increase peaking at about 5 s, followed by a undershoot. This region was not fully covered by our fNIRS montage and might have been missed by the fNIRS investigation.

**Figure 4:**
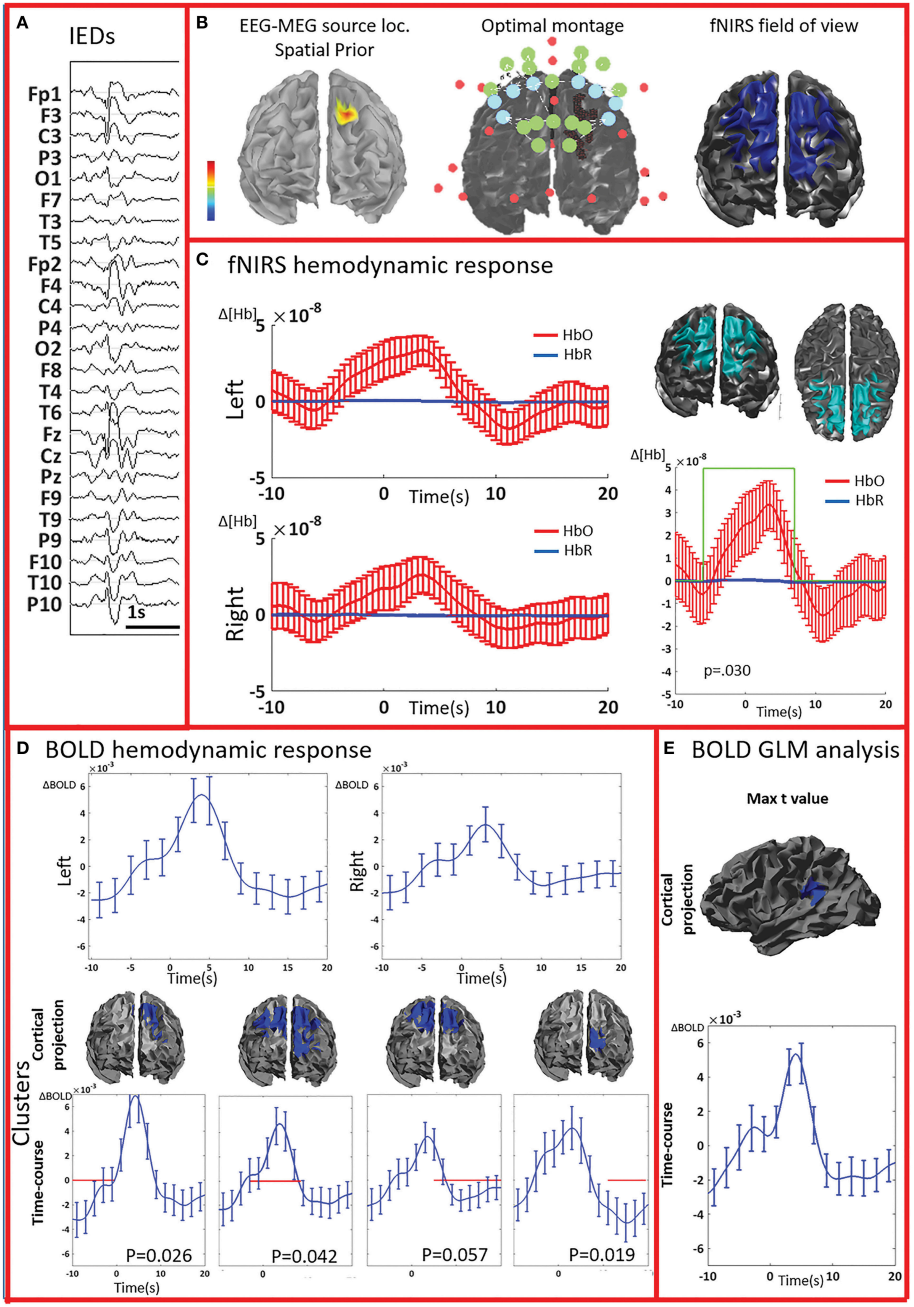
**Multimodal investigation of patient PA07**. Details regarding the organization of this figure are similar than those presented in Figure 2. **(A)** Spike and wave discharges bilaterally over the anterior regions, prevalent on the left side. **(B)** Left: EEG/MEG source localization performed at the first peak of the spike and wave discharge showed a left frontal generator. At the time of the wave complex, epileptic activity then propagated to both frontal regions. **(C)** An average hemodynamic response was detected on both sides, with slightly larger amplitude over the left/affected hemisphere. We then identified a large significant cluster involving both frontal areas characterized by a hemodynamic activity significantly different from the control events. The corresponding average hemodynamic response to IEDs of the cluster was of large amplitude starting earlier than the IEDs and peaking at about 5 s; a mild undershoot was present at about 10 s. **(D)** Average fMRI hemodynamic responses obtained over the fNIRS field of view exhibited bilateral BOLD changes associated to IEDs, also starting earlier than 0 s, peaking between 3 and 5 s, and of larger amplitude over the left/affected side. The cluster-permutation analysis unveiled a significant negative BOLD signal in the left frontal region starting at -10 s, followed by a marginally significant but large BOLD increase (*p* = 0.057 vs. control events) over both frontal regions. This is followed by the undershoot covering both frontal regions and ending over the left frontal areas, close to the epileptic focus. **(E)** The cluster exhibiting the maximum *t*-value found using standard GLM analysis was at the junction between the left temporal and pariental lobes. The corresponding BOLD response was canonical with an increase peaking at about 5 s, followed by a undershoot. This cluster is discordant with the results of EEG-MEG source localization and the BOLD response is of lower in amplitude compared to the one observed over the frontal regions. The standard GLM analysis did not provide any significant cluster in the left frontal region included in the field of view.

**Figure 5:**
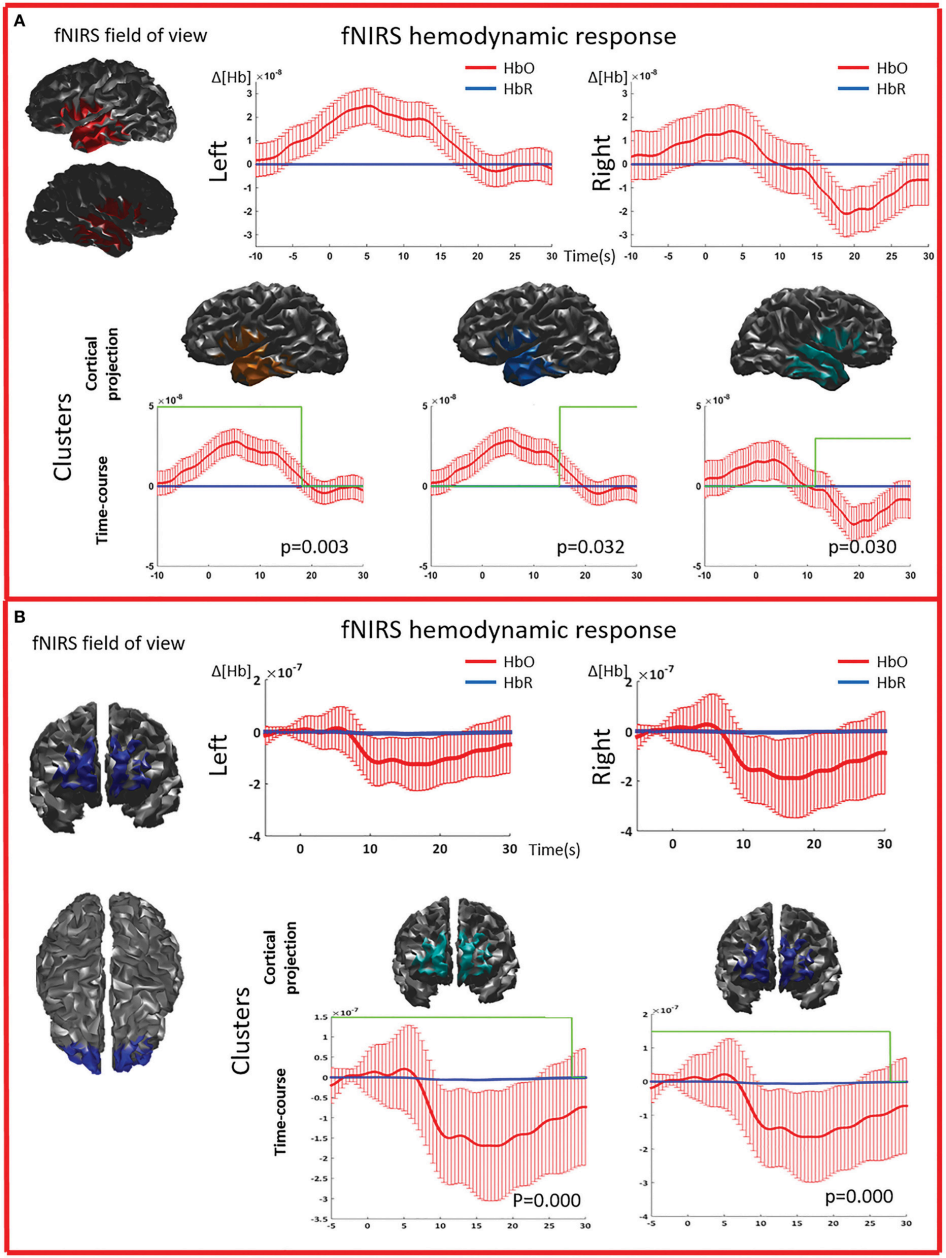
**Multimodal investigation of patient PA09 and PA03. (A)** PA09 with left temporal epilepsy. fNIRS averaged hemodynamic response with a clear HbO increase over the left/affected temporal region. An hemodynamic response was also found on the contralateral side, consisting mainly in a large decrease of HbO starting at about 10 s. This trend was further confirmed by cluster analysis. We identified two significant clusters over the left temporal regions showing respectively an HbO increase and a later HbO decrease, while over the right temporal region, the only significant cluster was exhibiting an HbO decrease. **(B)** Multimodal investigation of patient PA03 with right frontal epilepsy. fNIRS averaged hemodynamic response characterized by an initial small increase followed by a very intense bilateral HbO decrease, stronger over the right side. The cluster analysis confirmed such finding at the comparison with control markers.

Results summarizing our analysis of the fNIRS shape of the average hemodynamic response to IEDs are presented in Table 2. 8/9 patients (all but PA05) exhibited a HR to IEDs. In 7/8 cases (all but PA03) the HR was characterized by an overall HbO increase (HbO↑; Figures 2–5). In 6/8 patients we found an HbO↑ followed by an HbO↓ (PA02, PA03, PA04, PA05, PA07, PA09). One patient showed only an HbO↑ (PA01) preceded by an initial dip (HbO↓). PA08 was the only one showing an inverse response, with an HbO↓ followed by HbO↑. For PA03, HbO↑ was very brief and the following HbO↓ was very pronounced (Figure 5B). The HR started before 0 s for 3 patients (PA07, PA08, PA09; Figures 4, 5). The onset ranged between about -10 s (PA09) to about 5 s (PA4; median = 0 s). The overall duration (including both HbO increase and HbO decrease) ranged between 8.5 s (PA04) and about 30 s (PA01, PA09, PA06; mean = 21.94 s, standard error = 3.23 s). For 6/8 patients the hemodynamic response was lateralized, i.e., exhibiting either larger amplitude (PA01, PA02, PA07, and PA09) or longer duration (PA02 and PA04) on the side of the IEDs.

**Table 2:** **Summary of findings**.

**PA**	**EEG-NIRS**													**Affected Side (Cluster based)**	**Unaffected side**	**GLM cluster**
	**IEDs (number)**	**HR**	**HbO**	**Affected side**					**Unaffected**							
				**Shape description**	**Onset (s)**	**Peak (s)**	**End (s)**	**Overall duration (s)**	**Shape description**	**Onset (s)**	**Peak (s)**	**End (s)**	**Overall duration (s)**			
PA01	7	YES	↑	HbO initial dip (HbO ↓) at about 0 s, followed by HbO ↑	0	20	35	35	Initial dip (HbO ↓) with lower amplitude than affected side at about 0 s, followed HbO ↑	0	17.5	35	35	BOLD ↑ Onset = −6 s; Peak = 3 s; End = 8 s, followed by BOLD↓ Onset = 8 s, Nadir = 13 s, End = 24.5 s	BOLD ↑ Onset = −7 s, Peak = 2.5 s, End = 7.5 s, followed by BOLD ↓ Onset = 7.5 s, Nadir = 14 s, End = 25.5 s	BOLD ↓ Onset = −9 s; Nadir = −7 s; End = −5 s followed by BOLD ↑ Onset = 0.5 s; Peak = 4.5 s; End = 9 s. Left Occipital
PA02	209	YES	↑	HbO ↑followed by HbO↓	0	3.8	11	15	HbO ↑ with lower amplitude compared to the affected side	0	2.5	5	5	Non canonical and noisy	Non canonical and noisy	BOLD ↑ Onset = 0 s, Peak = 4.5 s, End = 8 s followed by BOLD ↓ Onset = 8 s, Nadir = 12.5 s, End = 22 s. Right temporal pole
PA03	32	YES	↑	Small HbO↑ at about 0 s followed by HbO ↓	7	17	20	20	small HbO↑ at about 0 s followed by HbO ↓;	7	17	20	20	–	–	–
PA04	15	YES	↑	HbO↑ followed by HbO↓	5.5	8.5	14	8.5	HbO ↓	0	2	4	4	–	–	–
PA05	>900	NO	–	–	–	–	–	–	–	–	–	–	–	–	–	–
PA06	22	YES	↑	HbO ↑ followed by HbO decrease	0	4.5	30	30	HbO ↑ followed by HbO decrease	0	6	35	35	BOLD ↑ Onset = −0.5 s; Peak = 2.5 s; End = 3.5 s, followed by BOLD↓; Onset = 3.5 s; Nadir = 6 s; End = 8 s	BOLD ↑ Onset = −3 s; Peak = −0.5 s; End = 1.5 s, followed by BOLD↓; Onset = 1.5 s; Nadir = 13 s; End = 21 s	BOLD ↑ Onset = −0.5 s; Peak = 2.5 s; End = 4.5 s followed by BOLD ↓ Onset = 4.5 s; Nadir = 6.5 s; End = 8.5 s. Right frontal
PA07	58	YES	↑	HbO ↑ followed by HbO decrease	−5	3.5	17.3	22.3	HbO ↑, weaker than on the affected side	−4.5	3.5	17.5	22	BOLD ↑ Onset = −4 s; Peak = 4 s; End = 8, followed by BOLD↓; Onset = 8 s; Nadir = 15.5 s; End = 23 s	BOLD ↑ Onset = −4.5 s; Peak = 3 s; End = 7 s, followed by BOLD↓; Onset = 7 s; Nadir = 10 s; End = 23 s	BOLD ↑ Onset = −5 s; Peak = 4 s; End = 8 s followed by BOLD ↓ Onset = 8 s; Nadir = 10.5 s; End = 24 s. Parieto-occipital junction
PA08	10	YES	↑	HbO ↓ followed by HbO↑	−4	5	10.7	14.7	HbO ↓ followed by HbO↑	−3.6	7.5	11.5	15	–	–	–
PA09	475	YES	↑	HbO ↑	−9.5	5	20.5	30	HbO ↑	−19	0	15	34	–	–	–

#### fNIRS cluster analysis

The cluster permutation analysis unveiled spatio-temporal clusters of hemodynamic response significantly different from control markers for 6/8 patients (PA01, PA02, PA03, PA06, PA07, PA09). They were found in both the affected and unaffected hemispheres (all cases except PA02). Since the cluster analysis was spatio-temporal, similar spatial regions could be represented by several clusters exhibiting a significant response at different time peaks. Indeed, multiple spatio/temporal clusters could be found in the same side for some patients (PA01, PA03, PA09).

### EEG-fMRI

#### EEG-fMRI standard analysis

All patients included in this study were screened for EEG-fMRI during their presurgical evaluation at Montreal Neurological Institute. PA09 was not scanned because of low spiking rate at telemetry. 3/8 patients did not show enough IEDs in the scanner to perform an fMRI analysis (PA03, PA04, PA08). PA05 showed unreliable results, with multiple, small and scattered clusters. For four cases (PA01, PA02, PA06, PA07) the region showing the highest *t*-value after standard GLM analysis corresponded to a lateralized and well-defined activation, which was concordant with the spike field for three cases (PA01, PA02, PA06) and discordant for the remaining one (PA07).

#### BOLD time course and cluster analysis

The analysis of the average BOLD time-course in the fNIRS field of view showed a hemodynamic response for 3/4 patients (PA01, PA06, and PA07; Table 1; Figures 2,4. In all these three cases the hemodynamic response was (i) bilateral, (ii) characterized by BOLD↑ followed by BOLD↓ (iii) starting earlier than the IEDs (iv) lasting from 8 to 30 s. We also evaluated the BOLD time course in the brain region corresponding to the cluster showing the highest *t*-value according to standard GLM analysis, i.e., where we would have expected to find the largest and clearest BOLD change. PA02 (Figure 3) showed indeed a very strong BOLD response with a canonical shape in this cluster. However, for PA06, despite a large, strong and clear activation with high *t*-value, the corresponding average BOLD time-course was quite noisy and non-canonical. Surprisingly, for PA01 (Figure 2) and PA07 (Figure 4), the BOLD change found in the main region identified by standard GLM analysis was lower in amplitude than the one found in the fNIRS field of view, suggesting that the GLM-based approach, assuming a fixed hemodynamic response, was underestimating the regions involved in the hemodynamic changes elicited by IEDs. The permutation analysis applied to fMRI data further confirmed these findings and unveiled for PA01 and PA07 spatio/temporal clusters of definite BOLD changes similar or larger in amplitude to those occurring in the region corresponding to the GLM-based cluster with the maximal *t*-value (Figures 2, 4).

### Illustrative cases

We report detailed illustrative examples for two selected patients. Note that further details for all the included patients can be found in the supplementary material.

PA01 (Figure 2) with left occipital epilepsy had EEG-MEG sources within the left posterior quadrant region. An optimal fNIRS montage was designed to cover this area bilaterally (Figure 2A). The averaged fNIRS response obtained from seven EEG markers (bursts of rhythmic fast activity) was characterized by an initial HbO dip (small decrease) over the affected side followed by a bilateral long-lasting HbO increase, exhibiting larger amplitude on the affected side. After fNIRS permutation analysis, significant fNIRS clusters were found on both hemispheres but the strongest response was located over the presumed focus and showed an initial dip. In the fNIRS field of view, the average BOLD signal obtained from 15 IEDs depicted a definite non-canonical, long-lasting hemodynamic response, starting earlier than the IEDs. This behavior was confirmed by the permutation analysis showing significant non-canonical clusters of BOLD increase followed by BOLD decrease on both sides. The BOLD response in the region exhibiting the highest *t*-value based on standard GLM analysis was obviously more canonical, but surprisingly with lower amplitude than in the regions identified by the cluster analysis. Overall, the hemodynamic response involves a broader region than the one unveiled by GLM analysis and there is a good spatial overlap between the fNIRS and fMRI clusters obtained from permutation analysis.

PA07 (Figure 4) had left frontal epilepsy with frequent, bilateral spike and wave discharges, maximum over the left side. EEG-MEG sources at the time of the spike of the spike and wave complex revealed a left frontal generator, which was used as spatial prior for the patient specific fNIRS optimal montage (Figure 4B). The averaged fNIRS response obtained from 58 IEDs was characterized by an HbO increase starting before the IEDs and of larger amplitude over the affected side. This is in agreement with the bilateral distribution of the spike and wave complex. The fNIRS permutation analysis identified only one significant cluster, covering a large bilateral frontal region, peaking at 5 s and starting earlier than the IEDs. There was a mild non-significant undershoot. The averaged BOLD signal to 31 IEDs, estimated in the fNIRS field of view, also showed a bilateral increase, peaking between at 3–5 s and starting earlier than the IEDs. Interestingly the permutation analysis showed that the BOLD changes started with a decrease originating in the affected side, very close to the source identified by EEG-MEG source localization and from where the discharges probably propagate. From there, the following BOLD increase involved both hemispheres, but the undershoot was definitely stronger on the left side. Finally, the cluster with the highest *t*-value obtained from standard GLM analysis was discordant with EEG-MEG source localization and located in the left parieto-temporal junction (no significant GLM analysis clusters found in the fNIRS field of view). This BOLD response was also discordant with the EEG spike field. In this region, the BOLD time-course was obviously canonical but of lower in amplitude than the one we found in the frontal regions.

## Discussion

Our main findings were that: (i) EEG-fNIRS is a suitable non-invasive technique to investigate the HR to IEDs (ii) the HR of IEDs is characterized by long-lasting HbO response, often bilateral, variable in shape across patients, (iii) thanks to prolonged acquisition time, EEG-fNIRS has greater chance to detect IEDs and associated hemodynamic responses when compared to EEG-fMRI investigations, (iv) our proposed cluster/permutation approach applied to fNIRS and fMRI data unraveled additional HR features underestimated when imposing a canonical HRF, i.e., non-canonical HR shape and involvement of larger brain areas.

### EEG-NIRS is a suitable non-invasive bedside technique to investigate the hemodynamic response to IEDs

In the last years, there has been an increasing interest in the potential clinical application of fNIRS for the study of epileptic disorders (Obrig, 2014). The majority of simultaneous EEG/fNIRS investigations have focused on the analysis of seizures (Villringer et al., 1994; Steinhoff et al., 1996; Adelson et al., 1999; Sokol et al., 2000; Watanabe et al., 2000, 2002; Haginoya et al., 2002; Buchheim et al., 2004; Munakata et al., 2004; Gallagher et al., 2008; Nguyen et al., 2012, 2013; Slone et al., 2012; Sato et al., 2013; Seyal, 2014; Yucel et al., 2014) or prolonged bursts of epileptic activity (Roche-Labarbe et al., 2008). Despite an overall small cumulative sample size (about 150–200 patients studied in the last 20 years), EEG-fNIRS has been able to show the HR to different types of seizures, which could not have been characterized otherwise. As previously mentioned, seizures are much less frequent than IEDs, and applying fNIRS to study interictal activity would allow a wider clinical diffusion of this technique.

Peng and collaborators have recently applied EEG-fNIRS to study the activation/deactivations related to IEDs. They showed a relatively low fNIRS sensitivity and specificity when using a GLM-based analysis (Peng et al., 2014). If, on the one hand, this study supports the idea that fNIRS can detect the HR to IEDs, it also suggests that further methodological improvements are needed to achieve higher sensitivity/specificity and a more accurate description of the HR shape. Following a different strategy, setting up a personalized multimodal approach and restricting fNIRS investigation to brain regions already known to be involved in the epileptic process, we found a significant fNIRS HR time-locked to IEDs for 8/9 consecutive patients. The response was often bilateral and non-canonical, implying that a more standard GLM approach, based on imposing a canonical hemodynamic response shape, would have probably underestimate the fNIRS performance.

The second step of our analysis consisted in testing the specificity of our results toward control events. The cluster/permutation approach allowed us identifying at least one significant spatiotemporal cluster for 6/8 patients. As already mentioned, this clustering/permutation method is quite conservative and some clusters of significant activity could still have been missed. On the other hand, we have good confidence that the ones detected were not false positives. The two patients who did not show significant clusters were PA08, who had been scanned for a shorter time, and PA04 who had a relatively lower signal quality, which might have affected our results. Multiple factors may influence the comparison between IEDs and control events, including the variability of the responses to IEDs as well as the ongoing physiological hemodynamic fluctuations associated to respiration and vasomotion (Jasdzewski et al., 2003; Franceschini and Boas, 2004; Hoshi, 2005). Moreover, control events were randomly extracted from periods free from any detectable epileptic activity from scalp EEG data. However, scalp EEG is only sensitive to some of the epileptic activity and will only detect discharges when the underlying cortical generator is extended over several square centimeters (Tao et al., 2007a,b; Von Ellenrieder et al., 2014). In other words, it cannot be ruled out that some of the control events might still be contaminated by HR to epileptic activity not visible on scalp EEG. In a similar line, previous studies have demonstrated EEG-fMRI HR even in the absence of scalp IEDs (Grouiller et al., 2011) and in few cases, fNIRS was suggested to exhibit better sensitivity to seizures than scalp EEG itself (Nguyen et al., 2013). Finally, it is worth highlighting that one single patient—PA05—did not show any HR after either EEG-fMRI (both standard analysis and cluster/permutation approach) or EEG-fNIRS analysis. This patient was showing a very high spiking rate, characterized by almost continuous low amplitude discharging. Therefore, we speculate that both standard GLM and cluster/permutation analyses were unable to contrast activity following the discharge to almost inexistent baseline periods.

### HR to epileptic discharges: Long-lasting HbO responses, often bilateral, highly variable across patients

#### Timing and shape of the hemodynamic response

Our study suggests that the HR to IEDs can be very long, with an average duration of about 24 s, ranging between 10 and 38 s, for IEDs that were only lasting few hundreds of milliseconds. Are such long HRs compatible with very brief epileptic events? We will report here five lines of evidence suggesting that a prolonged HR to IEDs is plausible.

First, previous fNIRS investigations on seizures have already shown a strong mismatch between the duration of electrical activity and the HR, consisting in much longer HR to seizures (Watanabe et al., 2000; Buchheim et al., 2004; Kobayashi et al., 2006c; Gallagher et al., 2008; Roche-Labarbe et al., 2008; Nguyen et al., 2013; Yucel et al., 2014). Notably, Haginoya et al. (2002) have shown that epileptic spasms, which are very brief epileptic seizures similar in duration to IEDs, are associated to hemodynamic effects lasting up to 50 s. Second, EEG-fMRI studies on IEDs suggest that the HR can last up to 31.5 s (Salek-Haddadi et al., 2006). Third, in humans, non-invasive transient depolarization of small neuronal pools obtained using single pulse transcranial magnetic stimulation (TMS) is associated to a fNIRS HR lasting more than 20 s (Thomson et al., 2011a,b, 2013). Forth, invasive studies, despite some controversies and the difficulty to extend those results to human non-invasive investigations (Wallois et al., 2010), confirmed that the HR can last much longer than the IEDs itself (Suh et al., 2006; Ma et al., 2009) and can start up to 6 s before the epileptic discharge (Osharina et al., 2010). Fifth, in the present study, similar results were also unveiled by the EEG-fMRI cluster/permutation analysis. Previous EEG-fMRI studies suggest that the duration of the HR can be influenced by the type of interictal discharge (Levan et al., 2010). A qualitative analysis of our results would suggest that the longest HR would correspond to patients showing either transient bursts of rapid activity or spike and wave complexes. However, our limited sample size prevents us from further interpretations. This interesting topic will be investigated in future studies.

About half of the patients showed a HR starting earlier than the IEDs itself. This is not surprising and in agreement with previous studies on animal model (Osharina et al., 2010) and EEG-fMRI (Hawco et al., 2007; Jacobs et al., 2009; Pittau et al., 2011; Benuzzi et al., 2012). Our analysis of the shape of the HR revealed increase of HbO as the most striking feature. This has been also underlined in previous EEG-fNIRS studies on ictal data (Gallagher et al., 2008; Nguyen et al., 2012, 2013; Yucel et al., 2014) and is likely mainly caused by a large increase of blood volume compensating the higher metabolic demand elicited by the epileptic discharge. For one patient (PA01), an initial “Dip” (Figure 2) was clearly identified, notably stronger over the presumed affected side and characterized by a transient decrease of HbO (Gallagher et al., 2008).

#### Spatial features of the hemodynamic response to IEDs

HR to IEDs was often found bilaterally, usually with larger amplitude over the affected side. For some patients the bilateral involvement was in agreement with documented bilateral epileptic activity observed by other modalities. For example, PA01 showed bilateral IEDs on EEG telemetry (Figure 2). PA03, PA06, and PA07 (Figure 4) exhibited spike and wave complexes, for which the only lateralizing feature was the spike lasting only few milliseconds, whereas the slow wave involved bilateral regions. Bilateral HR during epileptic discharges might be related to multiple factors, including spread of the epileptic activity, independent focus, differential spatial specificity between electrical and vascular response. All these mechanisms are well documented on animal model studies (Schwartz and Bonhoeffer, 2001), EEG-fNIRS investigations of seizures (Nguyen et al., 2012, 2013) and EEG-fMRI assessment of IEDs (Kobayashi et al., 2006a; Gotman, 2008; Yu et al., 2009). Overall, our EEG-NIRS results unveiled patterns of HR quite variable across patients, both in terms of time course and laterality. In the light of previous EEG-fNIRS and EEG-fMRI investigations, this suggests the need of studies on larger cohorts to better classify patterns of epileptic electrical activity and their vascular counterpart.

### EEG-NIRS has greater chance to detect IEDs and associated HR compared to EEG-fMRI

In this cohort, with standard EEG-fMRI analysis (Gotman, 2008), clusters of HR were observed only for half of the scanned patients, mainly because of the lack of spikes recorded in the scanner. Note that, on purpose, we did not select patients based on their fMRI findings, but rather on the focality of generators and EEG/MEG ability to localize a focus. This suggests that EEG-fNIRS can perform better in assessing the HR because prolonged acquisitions allow increasing the chances to collect IEDs. This effect depends on: (i) longer acquisitions, (ii) quiet environment facilitating drowsiness and sleep which, in turn, will maximize the chances for IEDs appearance (PA02, PA03, PA04, PA06, PA09). For four patients, we were able to compare the HR to IEDs measured by both EEG-fMRI and EEG-fNIRS investigations. fMRI analysis was performed in two different ways: (i) the standard approach (Gotman, 2008) based on a GLM followed by the identification of the cluster showing the highest *t*-value and (ii) a data-driven technique restricted to the fNIRS field of view using the cluster/permutation methodology of BOLD time courses proposed to analyze fNIRS data. This method allowed us to confirm significant BOLD response time-locked to IEDs, characterized by positive peak followed by an undershoot (PA01 and PA07, Figures 3, 4; Watanabe et al., 2014). However, there were two more striking findings. First, in agreement with our fNIRS results, the BOLD response can be larger and bilateral than the one identified using standard GLM approach (Figures 2, 4). Secondly, the average time-course of BOLD signals corresponding to the most significant cluster provided by standard GLM procedure was sometimes found noisy, non-canonical and of lower amplitude when compared to the BOLD response obtained from cluster/permutation analysis within the fNIRS field of view. Some authors have indeed recently underlined that the standard EEG-fMRI modeling might be at risk of underestimating the effect of epileptic events on brain's HR amplitude and distribution (Pittau et al., 2014). We verified this concept on both our EEG-fMRI and EEG-fNIRS data. A direct comparison between fNIRS and fMRI HR was beyond the scope of the present investigation and would require simultaneous fNIRS-fMRI recordings performed in a controlled environment which is the only strategy to capture the high correlation between BOLD and fNIRS signals (Strangman et al., 2002; Huppert et al., 2006; Cui et al., 2011).

### Methodological considerations

We have proposed an *ad-hoc* personalized strategy aiming at investing the fNIRS response to IEDs in patients with focal epilepsy. First of all, instead of trying to provide a complete spatial coverage of the full brain, we rather decided to accurately characterize the hemodynamic responses elicited by IEDs using a personalized optimal montage strategy (Machado et al., 2014). All the available sensors were dedicated to sample two regions: (i) the epileptic area, as identified by an EEG-MEG source localization, and (ii) its homologous contralateral region. Secondly, after using a neuronavigation system to carefully report on the subject's head the sensors positions of the optimal montage, we acquired fNIRS signals of good quality during prolonged periods by gluing the sensors to the scalp with collodion. In this study the acquisition was about four times longer than a standard EEG-fMRI, however this experimental set-up would be also suitable for recordings lasting up to days (Yucel et al., 2014).

This strategy provided the proper background to perform local 3D reconstruction of fNIRS activity. In order to estimate local fluctuations of HbO and HbR within each voxel of the gray matter, we applied the inverse reconstruction directly to both 690 and 830 nm signals, avoiding the need to estimate the differential path length when applying such conversion at the sensor level (Abdelnour et al., 2010). The forward model consisted in a realistic model of light propagation using Monte Carlo simulations through an anatomical model defined from the individual MRI (Machado et al., 2014). For the inverse reconstruction, we combined data from both 690 and 830 nm wavelengths within a minimum norm model, for which the amount of regularization was tuned using Restricted Maximum Likelihood. The amount of regularization was tuned on averaged data and then used to reconstruct the fNIRS response to each single IEDs and each control marker. In the tomographic approach, the spatial distribution of HbR and HbO changes can be described better, avoiding the exploration of multiple source-detector pairs. Moreover, the projection of the signal from sensors to brain attenuates sources of physiological noise, which are filtered out by the reconstruction algorithm itself (Boas et al., 2001). The 3D reconstruction might be quite demanding and not easily applicable in a clinical context but allows a more user-friendly visualization and interpretation of the results. Some of the most recent fNIRS software packages already allow these procedures which will be likely embedded in the software of fNIRS devices in a near future.

The data-driven statistical approach considered to detect spatio/temporal clusters of significant HR (Maris and Oostenveld, 2007), does not suffer from the temporal constraints usually imposed by GLM modeling (for a review see Tak and Ye, 2014). Consequently, our method allows to more easily unveil the temporal pattern of the HR associated to IEDs. This analysis was applied to HbO signals only, since HbO fluctuations are usually of larger amplitude and more reliable than HbR fluctuations (Strangman et al., 2002). The permutation analysis tested the specificity toward control events and was based on a metric which, relying on the sum of the absolute *t*-values of the voxels belonging to the cluster, accounts for both the intensity of the signal and dimension of the clusters (Maris and Oostenveld, 2007).

In this first study, we decided to simply represent average fNIRS responses to IEDs after the cluster based statistical analysis. We notably checked on realistic simulations that, even with some degree of temporal overlap, a simple average would be able to retrieve the main characteristics of the response. Future studies may want to explore the potential benefit of deconvolution techniques (Lina et al., 2010) to accurately recover the time course of the HR. Rather than using EEG/fNIRS as a localizing methodology, for which fMRI would of course provide better spatial resolution, we assume that our overall strategy is a necessary first step toward the set-up of a methodological framework to model and assess local neurovascular coupling processes at the time of these pathological discharges (Voges et al., 2012). This modeling was out of the scope of the present paper and will be investigated in future studies.

Albeit we used all the possible methodological and technological tricks to improve the accuracy of our investigation, this does not imply that in a daily clinical setting all these steps must be implemented. Further and larger studies would be needed to define the minimum requirements necessary for optimal fNIRS investigation in a clinical setting. In our experience the pre-planning of the experimental set-up with the estimation of an optimal montage, together with the use of a neuronavigation system to identify the positions of optodes and electrodes on the scalp, allowed an installation time much faster than a standard procedure.

The combination the assessment of fNIRS sensitivity together with an optimal montage allows to approach the clinical issue of fNIRS usefulness for generators located in deep brain regions, such as insula or longitudinal fissures. The low fNIRS sensitivity for these regions is a definite drawback of this technique compared to fMRI. Nonetheless, fNIRS preplanning might lead to a quantitative identification of those patients who would gain no benefit from this modality (including some patients who already had undergone brain surgery) and that should rather be assessed using EEG-fMRI.

In a clinical setting the advantage of using the individual MRI as opposed to a head template requires a specific assessment that we will address in a future study. Our present results show that the hemodynamic response to IEDs is often spatially smooth and diffuse and the burden associated with the personalization of the montage based on individual MRI might not be justified by the precision gained (Custo et al., 2010).

In conclusions, using a personalized EEG-fNIRS approach framed in a multimodal fashion and guided by EEG-MEG source localization, we detected the HR associated to IEDs, characterized their spatiotemporal features and their variability in time and space across subjects. The comparison with independently acquired EEG-fMRI data strengthened our confidence in fNIRS results, demonstrating that, because of longer acquisition time, EEG-fNIRS can outperform EEG-fMRI in detectability and ability to accurately recover the shape of the underlying HR to IEDs. On these bases, additional studies on larger cohorts will be needed to further validate the potential of this technique in the assessment of the neurovascular coupling in patients affected by drug resistant epilepsy.

## Author contributions

GP: study design, patients recruitment, data acquisition, analysis, and interpretation, manuscript preparation; AM: study design, data acquisition and analysis and interpretation. NE: data analysis and interpretation; SW: patients recruitment, data analysis; JH: study design, patients recruitment; JL: data analysis; EK: study design, patients recruitment, data acquisition and interpretation, manuscript preparation; CG: study design, data analysis and interpretation, manuscript preparation.

## Funding

GP is supported by Richard and Edith Strauss Canada Foundation. AM is supported by the Industrial Innovation Scholarships from the Fonds Québécois de la recherche sur la nature et les technologies (FRQNT), NSERC, and the Rogue Research company (Montréal, Canada) joint program. The whole project is supported by an NSERC Discovery and Discovery Accelerator Supplement grants as well as by a CIHR MOP 133619 from CG, CIHR MOP 93614 from EK; whereas the EEG/fNIRS acquisition system was acquired thanks to a grant from the Canadian Foundation for Innovation. Authors have no conflict of interest to disclose.

### Conflict of interest statement

The authors declare that the research was conducted in the absence of any commercial or financial relationships that could be construed as a potential conflict of interest.

## Footnotes

^1^http://neuroimage.usc.edu/brainstorm/Tutorials/TutBEst.

## Appendix A: EEG/MEG data acquisition and analysis

Simultaneous EEG/MEG data were recorded using a CTF-MEG-system (MISL, Vancouver, Canada) with 275 MEG gradiometers and 54 EEG electrodes arranged on a cap according to the 10/20 system plus additional electrodes according to the 10/10 system (F1, FPZ, F2, AF7, AF3, AFZ, AF4, AF8, FT9, FC5, FC3, FC1, FCZ, FC2, FC4, FC6, FT10, C1, C2, CP5, CP3, CP1, CPZ, CP2, CP4, CP6, P9, P1, P2, P10, PO7, PO3, POZ, PO4, PO8—Easy-cap, Herrsching, Germany). Eye movements and heartbeat were monitored using a bipolar montage and dedicated electrodes. EEG electrodes position and the full headshape were digitized using a Polhemus 3D localizer (Colchester, NH). The EEG/MEG acquisition lasted about 1 h and was performed in a quiet shielded room, with the patient lying down in a supine position. The sampling frequency was 1200 Hz and the head position inside the MEG helmet was continuously monitored using three localization coils placed on anatomical landmarks. EEG signals were re-referenced offline to bipolar and/or average montage and EEG and MEG data were visually inspected and marked using the DataEditor software (MISL, Vancouver, Canada) by two trained neurologists (G.P. and E.K.). The IEDs were marked at their peak. Offline data processing was performed in Brainstorm (Tadel et al., 2011) and included: third-order spatial gradient noise cancelation, DC removal, 60-Hz Notch filter, bandpass filter between 0.3 and 70 Hz, downsampling to 600 Hz, bad channels removal, EEG re-reference to average. BrainVISA-4.2.1 software (http://brainvisa.info/; Mangin et al., 1995) was used to segment the individual MRI and to reconstruct the surfaces of the skin and of the gray–white matter interface. The 3T individual MRI, the skin surface and a cortical mesh tessellated from the gray–white matter interface were imported in the Brainstorm software (Tadel et al., 2011) where the subsequent processing was performed. We reconstructed individual three-layers-BEM-surfaces (inner-skull, outer-skull, and skin) and co-registered anatomical and functional data applying a surface fitting between the anatomical head shape derived from the MRI and the head points digitized using the Polhemus system at the time of the EEG/MEG scan. The forward model, assessing the contribution of each dipolar source on all EEG and MEG sensors, was computed applying OpenMEEG Boundary Element Method (BEM; Gramfort et al., 2010) and more specifically its implementation in Brainstorm, using a one-layer boundary element model for MEG (conductivity: 0.33 S/m) and a three-layers BEM model for EEG (conductivity: brain 0.33 S/m, skull 0.165 S/m, and skin 0.33 S/m; brain-to-skull ratio: 1/20). IEDs and baseline MEG/EEG data were imported into Brainstorm as segmented signals (−200 to 600 ms, 0 ms being the IEDs peak; 2 s baseline). The inverse problem of EEG and MEG source imaging was solved on averaged IEDs signals by applying the coherent Maximum Entropy on the Mean (cMEM), a method that has been carefully evaluated for its ability to recover the location and the spatial extent of IEDs sources (Grova et al., 2006a; Chowdhury et al., 2013; Heers et al., 2014, 2016). cMEM relies on a reference distribution which is constructed prior to the source imaging analysis. The reference distribution is based on a data-driven parcellization of the whole cortex. On each parcel, an index determining whether the parcel is contributing or not to the data is attributed. Based on the index scores, some parcels can be considered as inactive and be switched off during the source reconstruction, thus reducing the amount of false positive and increasing the spatial accuracy.

## Appendix B: EEG-fMRI data acquisition and standard GLM analysis

The acquisition was performed using a 3T MRI scanner (Trio, Siemens, Erlangen, Germany) together with an MR-compatible EEG system (BrainAmp; Brain Products, Munich, Germany; 5 kHz sampling) with 25 EEG electrodes. The acquisition of anatomical data (1-mm slice thickness, 256 × 256 matrix, *TE* = 9.2 ms, repetition time *TR* = 22 ms, flip angle 30 degrees) was followed by BOLD fMRI for an average time of about 1 h. Scanner artifacts were removed using Brain Vision Analyzer (Brain Products GmbH; Allen et al., 2000), while the ballistocardiogram was removed using independent component analysis (Benar et al., 2003). IEDs similar to those recorded at telemetry/EEG-fNIRS/EEG-MEG were marked by two neurologists (S.W. and G.P.). Timing and duration of IEDs were used to build regressors and convolved using four hemodynamic response functions (HFRs) peaking respectively 3, 5, 7, and 9 s after the discharge (Bagshaw et al., 2004). fMRIStat software (Worsley et al., 2002) was used to generate statistical t-map for each regressor. Then, at each single voxel, the maximal *t*-value obtained from modeling each delayed HRF was taken to generate a “combined” t-map (Bagshaw et al., 2004) which was thresholded at |*t*| > 3.1 with a minimum cluster spatial extent of five voxels (corrected family-wise error *p*-value = 0.05). This procedure allowed identifying regions of significant activation/deactivations related to epileptic events, but assuming a canonical time-course of the hemodynamic response to epileptic activity.
