# Supplementary material for: Hemodynamic Response to Interictal Epileptiform Discharges Addressed by Personalized EEG-fNIRS Recordings
Source: Front Neurosci. 2016 Mar 22;10:102. doi: 10.3389/fnins.2016.00102 (PMC4801878; doi:10.3389/fnins.2016.00102)
Supplement: Supplementary file 1 [file DataSheet1.docx]

Detailed description of single subject results

**PA01**

*CLINICAL NOTES:* PA01 is a 44 years old female affected by left occipital focal epilepsy. She presents very brief Complex Partial Seizures, very brief. The frequency is 1-2 per week on average, but occasionally presents cluster of up to 10 seizures per day. The anatomical MRI is normal. EEG telemetry is characterized by posterior quadrant IEDs (spikes, sharp-waves and brief burst of fast activity), synchronous and asynchronous on both sides, more frequent on left side.

*MULTIMODAL INVESTIGATION:* The simultaneous EEG-MEG scan mainly showed brief bursts of rapid activity over the posterior quadrant regions on both sides, prevalent on the left. Source localization identified bilateral occipital sources (lateral occipital gyrus), exhibiting larger amplitude on the left side. The standard GLM EEG-fMRI analysis of brief bursts of rapid activity over the left occipital cortex showed a maximal cluster of activation corresponding to the left occipital region. However, albeit with lower t-values, other clusters were found on both sides. Overall, EEG-MEG and EEG-fMRI showed concordant results.

*OPTIMAL MONTAGE:* targeting left lateral occipital gyrus.

*EEG-NIRS*

- *Parameters:* Acquisition time 4 hours. IEDs analyzed: burst of rapid activity. Time constrain for isolated events: 35s. Number of IEDs events: 7. Number of control events: 87.
- *Results – spatio-temporal dynamics - :*
  - *Overall hemodynamic response*
    - *Affected (left) side:* HbO initial dip (HbO ↓) at about 0s, followed by HbO ↑ onset= 2s; peak=20s; End= 35s.
    - *Unaffected (right) side:* initial dip (HbO ↓) with lower amplitude when compared to the affected side, at about 0s, followed HbO ↑ Onset=6.5s; Peak=17.5s; End=35s. Possible initial dip, not as clear as for Affected (left) side.
  - *Permutation clusters*
    - Cluster 1. Side: unaffected (right); HbO ↓; Onset=-2.55s; End=2.1s; p=0.039
    - Cluster 2. Side: affected (left); HbO ↑; Onset=9.95; End=15.25; p=0.018
    - Cluster 3. Side: unaffected (right); HbO ↑; Onset=10.95; End=21.6s; p=0.022
    - Cluster 4. Side: unaffected (right); HbO ↑; Onset=21.95; End=29.5; p=0.014

*EEG-fMRI*

- *Parameters:* Acquisition time 1 hour. IEDs analyzed: burst of rapid activity. Time constrain for isolated events: 15s. Number of IEDs events: 15. Number of control events: 163.
- *Results – spatio-temporal dynamics -:*
  - *Overall BOLD response*
    - *Affected (left) side:* BOLD ↑ Onset=-6s; Peak=3s; End=8s, followed by BOLD↓ Onset=8s, Nadir=13s, End=24.5s
    - *Unaffected (right) side:* BOLD ↑ Onset=-7s, Peak=2.5s, End=7.5s, followed by BOLD ↓ Onset=7.5s, Nadir=14s, End=25.5s
  - *Permutation clusters*
    - Cluster 1. Side: affected (left); BOLD ↑; Onset=-4.5s; End=9.5s; p=0.011
    - Cluster 2. Side: unaffected (right); BOLD ↑; Onset=-4.5s; End=9s; p=0.005
    - Cluster 3. Side: unaffected (right); BOLD ↓; Onset=10s; End=17.5s; p=0.028
    - Cluster 4. Side: affected (left); BOLD ↓; Onset=9.5s; End=18s; p=0.012
  - Standard GLM EEG-fMRI analysis
    - Max t-value cluster: affected side.
      - BOLD ↓ Onset=-9s; Nadir=-7s; End=-5s
      - BOLD ↑ Onset=0.5s; Peak=4.5s; End=9s

*Additional notes and comments:* the fNIRS hemodynamic response seems to involve a smaller region over the affected (left) side compared to the contralateral. This impression might be biased by the lower number of sensors available on the left side after discarding those affected by artifact which makes the field of view different on the two sides.

**PA02**

*CLINICAL NOTES:* PA02 is a 25 years old female, affected by right neocortical temporal epilepsy. She presents Complex Partial Seizures with an average frequency of 2-3 per month and rare Generalized Tonic Clonic Seizures. Four years ago, she showed a right frontal epileptic focus whose activity was studied at both telemetry and EEG-MEG. Such epileptic focus has not been active during the past three years. The anatomical MRI is normal. EEG telemetry shows IEDs over the right temporal regions, characterized by Spike and wave complexes and, mainly during sleep, bursts of fast activity.

*MULTIMODAL INVESTIGATION:* EEG-MEG mainly recorded spike and wave complexes over the right temporal region whose source localization showed a right temporal source (middle temporal gyrus). A previous EEG-MEG performed 4 years earlier had shown right frontal spike and wave complexes whose source localization had shown a right frontal generator. Standard EEG-fMRI with GLM analysis of right temporal spike and wave complexes showed an activation with maximal t-value in the right temporal lobe (anterior aspect of right middle and superior temporal gyra), including the right temporal pole.

*OPTIMAL MONTAGE:* targeting the right temporal lobe. Additional target region was in the right frontal lobe, as assessed by the previous EEG-MEG performed 4 years earlier.

*EEG-NIRS*

- *Parameters:* Acquisition time 4 hours. IEDs analyzed: spike and wave complex. Time constrain for isolated events: 5s. Number of IEDs events: 209. Number of control events: 69.
- *Results –* spatio-temporal dynamics-:
  - *Overall hemodynamic response*
    - *Affected (right) side:* HbO ↑; Onset=0s; Peak=3.75s; End=6.5s; followed by HbO↓; Onset=6.5s; End>10s.
    - *Unaffected (left) side:* HbO ↑ with lower amplitude compared to the affected side; Onset=0s; Peak=2.5s; End=5s.
  - *Permutation clusters*
    - Cluster 1: Side: affected (right), frontal region; HbO ↑; Onset=2s; End=5.3s; p=0.037

*EEG-fMRI*

- *Parameters:* Acquisition time 1 hour. IEDs analyzed: spike and wave complex. Time constrain for isolated events: 15s. Number of IEDs events: 29. Number of control events: 150.
- *Results – spatio-temporal dynamics -:*
  - *Overall BOLD response*
    - Non canonical and noisy
  - *Permutation clusters:* none
  - *Standard GLM EEG-fMRI analysis*
    - Max t-value cluster: right temporal lobe.
      - BOLD ↑ Onset=0s, Peak=4.5s, End=8s
      - BOLD ↓ Onset=8s, Nadir=12.5s, End=22s

*Additional notes and comments*: The fNIRS sensors over the left frontal region were discarded because of bad quality data. The BOLD time-course in the cluster with maximal t-value obtained by standard EEG-fMRI analysis shows a canonical response. This cluster involved the superior part of the anterior temporal lobe and the temporal pole and was partially overlapping with the EEG/MEG source. fNIRS field of view included this zone but being optimized on EEG-MEG did not properly explore the temporal pole.

**PA03**

*CLINICAL NOTES:* PA03 is a 24 years old female affected by right frontal epilepsy. She presents brief Complex Partial Seizures, up to many per day. The anatomical MRI is normal. EEG shows bursts of fast activity with right frontal and temporal predominance during sleep and bilateral and synchronous spike and Wave discharges with maximal amplitude over the frontal regions.

*MULTIMODAL INVESTIGATION:* EEG-MEG showed bilateral frontal spike and wave discharges and burst of rapid activity. Source localization applied to the spike of the spike and wave complexes suggested a right frontal involvement. EEG-fMRI has been performed twice but no IEDs have been recorded.

*OPTIMAL MONTAGE:* targeting the right frontal cortex

*EEG-NIRS*

- *Parameters:* Acquisition time 4 hours. IEDs analyzed: burst of rapid activity. Time constrain for isolated events: 15s. Number of IEDs events: 32. Number of control events: 124.
- *Results – spatio-temporal dynamics - :*
  - *Overall hemodynamic response*
    - *Affected (right) side:* small HbO↑ at about 0s followed by a large HbO ↓; Onset=7s; Nadir=17s; End>20s.
    - *Unaffected (left) side: similar response as on the affected side.*
  - *Permutation clusters*
    - Cluster 1. Side: bilateral; HbO ↓; Onset= ; Peak=; End=28.1; p=0.000
    - Cluster 2. Side: bilateral; HbO ↓; Onset= ; Peak=; End=28.1; p=0.000

*Additional notes and comments*: fNIRS overall shows a bifrontal small HbO increase, followed by a huge and long lasting bilateral HbO decrease.

**PA04**

*CLINICAL NOTES:* PA04 is a 53 years old male affected by right occipital epilepsy. He presents Complex Partial Seizures preceded by a visual aura. The frequency is in clusters, with up to many seizures per day. The anatomical MRI is normal. EEG shows sharp and slow waves over the right posterior region, usually during sleep. Occasionally, he also presents right temporal sharp waves.

*MULTIMODAL INVESTIGATION:* EEG-MEG showed sharp waves over the right occipital region whose source localization unveiled a clear right occipital focus involving the fusiform gyrus.

*EEG-fMRI*: performed twice but no IEDs have been recorded.

*OPTIMAL MONTAGE:* targeting the right occipital cortex

*EEG-NIRS*

- *Parameters:* Acquisition time 4 hours. IEDs analyzed: sharp waves over the right occipital regions. Time constrain for isolated events: 15s. Number of IEDs events: 51. Number of control events: 209.
- *Results – spatio-temporal dynamics - :*
  - *Overall hemodynamic response*
    - *Affected (right) side:* HbO↑; Onset=5.5s; Peak=8.5s; End=10.5s; HbO↓; Onset=10.5s; Nadir=12s; End=14s.
    - *Unaffected (left) side:* HbO ↓; Onset=0s; Peak=2s; End=4s.
  - *Permutation clusters:* none

**PA05**

*CLINICAL NOTES:* PA05 is a 28 years old male, affected by left parieto-occipital epilepsy. He presents occasional Complex Partial Seizures, usually preceded by visual aura and occasionally followed by secondary generalization. Seizure frequency is about 2 per year. The anatomical MRI is normal. EEG shows very frequent and low amplitude spikes over the left parieto-occipital junction.

*MULTIMODAL INVESTIGATION:* EEG-MEG showed left parieto-occipital spikes whose source localization unveiled a left parieto-occipital focus. During the standard EEG-fMRI were recorded many spikes but the pattern of activation/deactivation was scattered and noisy.

*OPTIMAL MONTAGE:* targeting the left parieto-occipital cortex.

*EEG-NIRS*

- *Parameters:* Acquisition time 4 hours. IEDs analyzed: more than 10000 low amplitude left parieto-occipital spikes. No evidence of hemodynamic response

*EEG-fMRI*

- *Parameters:* Acquisition time 1 hour. IEDs analyzed: many low amplitude left parieto-occipital spikes. Time constrain for isolated events: 15s. No evidence of hemodynamic response.

*Additional notes and comments*: Neither EEG-NIRS nor EEG-fMRI (both using standard GLM approach and cluster-permutation approach) showed a clear and significant hemodynamic response. We believe that multiple reasons explain such behavior: spike amplitude was very low and the spiking rate very high making likely a large contamination of focus-related hemodynamic response over the presumed baseline period considered to contrast the results.

**PA06**

*CLINICAL NOTES:* PA06 is a 28 years old female affected by right frontal lobe epilepsy. She presents complex partial seizures characterized by head version to the left. The frequency is about 1 seizure per month. The anatomical MRI is non-lesional. EEG telemetry shows bilateral and synchronous frontal spike and wave discharges, often prevalent in amplitude on the right side, sometimes organized in sequences lasting a few seconds.

*MULTIMODAL INVESTIGATION:* The simultaneous EEG-MEG scan shows frontal bilateral spike and wave complexes for which the spike source localization unveiled a right frontal focus. The standard EEG-fMRI analysis of frontal bilateral spike and wave complexes showed an activation with maximal t-value in the right frontal lobe, very widespread.

*OPTIMAL MONTAGE:* targeting the right frontal lobe.

*EEG-NIRS*

- *Parameters:* Acquisition time 4 hours. IEDs analyzed: spike and wave complex. Time constrain for isolated events: 10s. Number of IEDs events: 22. Number of control events: 45.
- *Results –* spatio-temporal dynamics-:
  - Overall hemodynamic response
    - Affected (right) side: HbO ↑; Onset=0s; Peak=4.5s; End=13.5s; This is then followed by HbO↓; Onset=14s; Nadir=30s; End>30s;
    - Unaffected (left) side: HbO ↑, weaker than on the affected side; Onset=0.2s; Peak=5.9s; End=11.3s. Delayed HbO↓; Onset= 25s; Nadir=31s; End>35s.
  - *Permutation clusters*
    - Cluster 1. Side: unaffected (left); HbO ↑; Onset=20.5s; End=25.2s; p=0.035
    - Cluster 2: Side: affected (right); HbO↓; Onset=26.5s; End=35s; p=0.046

*EEG-fMRI*

- *Parameters:* Acquisition time 1 hour. IEDs analyzed: spike and wave complex. Time constrain for isolated events: 15s. Number of IEDs events: 14. Number of control events: 65.
- *Results – spatio-temporal dynamics -:*
  - *Overall BOLD response*
    - Affected (right) side: BOLD ↑ Onset=-0.5s; Peak=2.5s; End=3.5s, followed by BOLD↓; Onset=3.5s; Nadir=6s; End=8s.
    - Unaffected (left) side: BOLD ↑ Onset=-3s; Peak=-0.5s; End=1.5s, followed by BOLD↓; Onset=1.5s; Nadir=13s; End=21s.
  - *Permutation clusters:* none
  - Standard EEG-fMRI analysis
    - Max t-value cluster: right frontal cortex
    - BOLD ↑ Onset=-0.5s; Peak=2.5s; End=4.5s
    - BOLD ↓ Onset=4.5s; Nadir=6.5s; End=8.5s

*Additional notes and comments*: in fMRI we did not find any clusters exhibiting a significant response when comparing IEDs and control markers. We believe that the very high spike rate observed for this patient might have biased the baseline/control periods and hamper the cluster-permutation procedure. The EEG-fNIRS because of longer acquisition allowed recording more epileptic events but also longer non epileptic periods.

PA07

*CLINICAL NOTES*: PA07 is a 21 years old female, affected by left frontal epilepsy. She presents Complex Partial Seizures without clear aura, often in clusters up to many per day. Anatomical MRI is normal. EEG telemetry shows bilateral and synchronous frontal spike and wave complexes, possibly prevalent over the left side.

*MULTIMODAL INVESTIGATION:* The simultaneous EEG-MEG scan showed frontal bilateral spike and wave complexes for which the spike source localization unveiled a left frontal focus. The standard EEG-fMRI analysis of frontal bilateral spike and wave complexes showed a very high t-value cluster in the left parieto-temporal junction.

*OPTIMAL MONTAGE:* targeting the left frontal lobe.

*EEG-NIRS*

- *Parameters:* Acquisition time 4 hours. IEDs analyzed: spike and wave complex. Time constrain for isolated events: 15s. Number of IEDs events: 58. Number of control events: 52.
  - Overall hemodynamic response
    - Affected (left) side: HbO ↑; Onset=-5s; Peak=3.5s; End=8.1s; this is then followed by HbO↓; Onset=8.1s; Nadir=11.5s; End=17.35s.
    - Unaffected (right) side: HbO ↑, weaker than on the affected side; Onset=-4.5s; Peak=3.5s; End=8.5s. This is then followed by HbO↓; Onset= 8.5s; Nadir=11s; End=17.5s.
  - *Permutation clusters*
    - Cluster 1. Side: bilateral; HbO ↑; Onset=-6s; Peak=3.15s; End=7s

*EEG-fMRI*

- *Parameters:* Acquisition time 1 hour. IEDs analyzed: spike and wave complex. Time constrain for isolated events: 15s. Number of IEDs events: 31. Number of control events: 45.
- *Results – spatio-temporal dynamics -:*
  - *Overall BOLD response*
    - Affected (left) side: BOLD ↑ Onset=-4s; Peak=4s; End=8, followed by BOLD↓; Onset=8s; Nadir=15.5s; End=23s.
    - Unaffected (right) side: BOLD ↑ Onset=-4.5s; Peak=3s; End=7s, followed by BOLD↓; Onset=7s; Nadir=10s; End=23s.
  - *Permutation clusters*
    - Cluster 1. Side: affected (left); BOLD ↓; Onset=-10s; End=-0.5s; p=0.026
    - Cluster 2. Side: bilateral; BOLD ↑; Onset=-3s; Peak=4s; End=8.5s; p=0.042
    - Cluster 3. Side: bilateral; BOLD ↓; Onset=5s; Nadir=10.5s; End=20s; p=0.057
    - Cluster 3. Side: bilateral; BOLD ↓; Onset=11s; End=19.5s; p=0.019
  - Standard EEG-fMRI analysis
    - Max t-value cluster: left parieto-occipital junction
      - BOLD ↑ Onset=-5s; Peak=4s; End=8s
      - BOLD ↓ Onset=8s; Nadir=10.5s; End=24s

**PA08**

*CLINICAL NOTES:* PA08 is a 29 years old female affected by left temporal epilepsy. She presents complex partial seizures with a frequency of about 4-6 per month. The MRI shows bilateral periventricular nodular heterotopia close to the posterior aspect of the lateral ventricles. EEG telemetry shows left fronto-temporal spikes.

*MULTIMODAL INVESTIGATION:* The simultaneous EEG-MEG scan showed left temporal spikes whose source localization unveiled a focus in the left temporal region. EEG/fMRI analysis: no spikes found during 1 hour recording.

*OPTIMAL MONTAGE:* The target region was in the left temporal lobe.

*EEG-NIRS*

- *Parameters:* Acquisition time 3 hours. IEDs analyzed: spikes. Time constrain for isolated events: 15s. Number of IEDs events: 10. Number of control events: 43.
- *Results –* spatio-temporal dynamics-:
  - Overall hemodynamic response
    - Affected (left) side: HbO ↓; Onset=-4s; Nadir=0s; End=5s; This is then followed by HbO↑; Onset=5s; Peak=7.5s; End=10.7s.
    - Unaffected (right) side: HbO ↓; Onset=-3.6s; Nadir=0.5s; End=5s; This is then followed by HbO↑; Onset=5s; Peak=7.5s; End=11.5s;
  - *Permutation clusters: none*

PA09

*CLINICAL NOTES:* PA09 is a 35 years old male affected by left temporal lobe epilepsy. He presents occasional secondary generalized tonic clonic seizure whose clinical onset is not very clear. The anatomical MRI is normal. EEG telemetry shows left fronto-temporal spikes, mainly during sleep.

*MULTIMODAL INVESTIGATION:* The simultaneous EEG-MEG scan shows left temporal spikes whose source localization unveiled a left temporal focus. EEG-fMRI was not performed because during wakefulness the patient is not active enough

*OPTIMAL MONTAGE:* targeting the left temporal lobe.

*EEG-NIRS*

- *Parameters:* Acquisition time 4 hours. IEDs analyzed: spikes. Time constrain for isolated events: 0s. Number of IEDs events: 475. Number of control events: 272.
  - Overall hemodynamic response
    - Affected (left) side: HbO ↑; Onset=-9.5s; Peak=5s; End=20.5s;
    - Unaffected (right) side: HbO ↑; Onset=-19s; Nadir=0.5s; End=15s;
  - *Permutation clusters:*
    - Cluster 1. Side: affected (left- temporal region); HbO ↑; Onset=-10s; End=18s; p=0.003
    - Cluster 2. Side: affected (left – temporal region); HbO ↓; Onset=15; End=35s; p=0.032
    - Cluster 3. Side: unaffected (right- temporal region); HbO ↓; Onset=11.5; End=35s; p=0.030
